# Oligotyping reveals differences between gut microbiomes of free-ranging sympatric Namibian carnivores (*Acinonyx jubatus, Canis mesomelas*) on a bacterial species-like level

**DOI:** 10.3389/fmicb.2014.00526

**Published:** 2014-10-14

**Authors:** Sebastian Menke, Wasimuddin, Matthias Meier, Jörg Melzheimer, John K. E. Mfune, Sonja Heinrich, Susanne Thalwitzer, Bettina Wachter, Simone Sommer

**Affiliations:** ^1^Evolutionary Genetics, Leibniz Institute for Zoo and Wildlife ResearchBerlin, Germany; ^2^Evolutionary Ecology, Leibniz Institute for Zoo and Wildlife ResearchBerlin, Germany; ^3^Institute of Vertebrate Biology, Academy of Sciences of the Czech RepublicBrno, Czech Republic; ^4^Department of Biological Sciences, University of NamibiaWindhoek, Namibia; ^5^Institute of Experimental Ecology, University of UlmUlm, Germany

**Keywords:** gut microbiome, bacteria, oligotyping, carnivores, cheetah (*Acinonyx jubatus*), black-backed jackal (*Canis mesomelas*), Namibia

## Abstract

Recent gut microbiome studies in model organisms emphasize the effects of intrinsic and extrinsic factors on the variation of the bacterial composition and its impact on the overall health status of the host. Species occurring in the same habitat might share a similar microbiome, especially if they overlap in ecological and behavioral traits. So far, the natural variation in microbiomes of free-ranging wildlife species has not been thoroughly investigated. The few existing studies exploring microbiomes through 16S rRNA gene reads clustered sequencing reads into operational taxonomic units (OTUs) based on a similarity threshold (e.g., 97%). This approach, in combination with the low resolution of target databases, generally limits the level of taxonomic assignments to the genus level. However, distinguishing natural variation of microbiomes in healthy individuals from “abnormal” microbial compositions that affect host health requires knowledge of the “normal” microbial flora at a high taxonomic resolution. This gap can now be addressed using the recently published oligotyping approach, which can resolve closely related organisms into distinct oligotypes by utilizing subtle nucleotide variation. Here, we used Illumina MiSeq to sequence amplicons generated from the V4 region of the 16S rRNA gene to investigate the gut microbiome of two free-ranging sympatric Namibian carnivore species, the cheetah (*Acinonyx jubatus*) and the black-backed jackal (*Canis mesomelas*). Bacterial phyla with proportions >0.2% were identical for both species and included Firmicutes, Fusobacteria, Bacteroidetes, Proteobacteria and Actinobacteria. At a finer taxonomic resolution, black-backed jackals exhibited 69 bacterial taxa with proportions ≥0.1%, whereas cheetahs had only 42. Finally, oligotyping revealed that shared bacterial taxa consisted of distinct oligotype profiles. Thus, in contrast to 3% OTUs, oligotyping can detect fine-scale taxonomic differences between microbiomes.

## Introduction

Gut-associated bacterial communities and their mammalian hosts are highly dependent on each other. Recent investigations have applied metagenomic approaches to increase our understanding of the factors that shape these host-gut bacterial relationships (Kau et al., 2011; Muegge et al., 2011; Schloissnig et al., 2012). The interpretation of these results is, however, challenging because several factors such as host-bacteria co-evolution (Ley et al., 2008; Ochman et al., 2010; Yeoman et al., 2011), host genotype (Benson et al., 2010; Spor et al., 2011; Bolnick et al., 2014), life history traits and behavior (Ezenwa et al., 2012), social organization (Koch and Schmid-Hempel, 2011), health status, diet (Turnbaugh et al., 2009) and the environment itself (Coolon et al., 2010; Nelson et al., 2013) are simultaneously involved in shaping the gut microbiome. In contrast, variations in the gut microbiome affect the host by causing, for example, obesity (Turnbaugh et al., 2006) and changes in exploratory behavior or anxiety (Bercik et al., 2011; Bravo et al., 2011), all of which may affect the overall health status of the host (Sekirov et al., 2010; Hooper et al., 2012).

Previous studies on gut microbiomes have focused largely on the variation exhibited in humans or laboratory organisms. Only recently the interest has grown to study host-gut bacterial associations also in wildlife species (Schwab et al., 2011; Amato et al., 2013; Nelson et al., 2013; Delsuc et al., 2014). Such studies are facing many challenges, but offer, when sample sizes are large, important insight in the “normal” variation of the gut microbiome of free-ranging species. Only under natural conditions we can also detect how changes in the mentioned factors affect bacterial communities and thus host nutrition and health (McKenna et al., 2008; Amato, 2013). In addition, such data may provide reference information on habitat quality which has many implications for species conservation.

Here, we present for the first time a comparison of the gut microbiomes of two sympatric free-ranging mammalian carnivorous species, represented by a felid (cheetah, *Acinonyx jubatus*) and a canid (black-backed jackal, *Canis mesomelas*). We investigated their gut bacterial diversity by applying high-throughput sequencing to characterize the V4 hyper-variable region of the 16S rRNA gene. Differences in bacterial composition between feline and canine species have been shown previously in domestic animals (Handl et al., 2011), but microbiomes in cheetahs have only been investigated in a few zoo individuals (Ley et al., 2008; Becker et al., 2014) and so far no study has investigated microbiomes in black-backed jackals. Thus, with our study we aim to provide the microbiomes of free-ranging cheetahs and black-backed jackals and to investigate hypotheses on bacterial diversity derived from known species characteristics.

We hypothesized that diet, foraging behavior, social system and home range size, four species characteristics that differ between the species, should also lead to differences in their gut-microbial diversities. The carnivorous diet of cheetahs (Eaton, 1974; Wachter et al., 2006) is likely to be associated with a lower microbial diversity than the omnivorous diet of black-backed jackals (Goldenberg et al., 2010), because the digestive requirements for a carnivorous species can be expected to be lower (Ley et al., 2008). Accordingly, cheetahs should harbor a lower gut-microbial community compared to black-backed jackals. Moreover, cheetahs feed only on freshly killed prey animals (Caro, 1994), not on carcasses as black-backed jackals occasionally do (Walton and Joly, 2003). An intake of a more diverse bacterial community due to scavenging can be expected and accordingly a lower microbial diversity in cheetahs than in black-backed jackals. Also, the intraspecific contact rate of mainly solitary cheetahs (Caro, 1994) is likely to be lower than the one of the group living black-backed jackals (Walton and Joly, 2003), resulting in a lower bacterial transmission and therefore an expected lower microbial diversity in cheetahs than in black-backed jackals. In contrast, the larger home range sizes of cheetahs (Marker et al., 2008) compared to black-backed jackals (Jenner et al., 2011; Kamler et al., 2012) are likely to result in cheetahs encountering a larger variety of environmental bacteria than black-backed jackals and therefore are expected to exhibit a higher microbial diversity. If the home range size has the stronger influence in shaping the microbiome of a host species, we expect the cheetah to exhibit a higher microbial diversity, but if diet, foraging behavior and social system have the stronger influence, we expect the black-backed jackal to exhibit a higher microbial diversity.

The taxonomic resolution of bacterial communities based on high-throughput sequencing of 16S rRNA gene amplicons is limited. The short fragment sizes, the single locus approach and the limitations in resolution and richness of most current databases hinders a taxonomic assignment of sequencing reads better than family or genus level. *De novo* clustering of reads into operational taxonomic units (OTUs) based on a similarity threshold (e.g., 97%, Caporaso et al., 2012) aims to increase the resolution. Nevertheless, multiple 3% OTUs can be assigned to a single genus and thus still contain unexplained diversity. Differences in bacterial communities between species, however, are manifested on a bacterial species or strain level (Suchodolski, 2011) and are therefore not detectable with the conventional OTU approach. Recently, this problem was addressed by Eren et al. (2013) who developed an “oligotyping” approach which reveals differences between bacterial communities on a low level of taxonomic discrimination by targeting subtle nucleotide variation. First publications that successfully applied oligotyping showed the potential of this method by having tracked human fecal *Lachnospiraceae* in sewage (McLellan et al., 2013) or having described the diversity of a single bacterial species in the genitourinary tract of monogamous sexual partners (Eren et al., 2011). If a comprehensive database is available, it is even possible to assign species level taxonomy to oligotypes (Eren et al., 2014).

In this study, we aim to investigate whether the diversity and proportion of bacterial taxa differ between the cheetah and the black-backed jackal and whether oligotype profiles within shared bacterial taxa are the same or not. To our knowledge, this is the first study that applies the new oligotyping approach in conjunction with the common OTU approach to describe the gut microbiome of sympatric carnivores using high-throughput sequencing of the 16S rRNA gene. This study contributes to our understanding on the extent to which host characteristics contribute to the variability of bacterial communities, from the bacterial phylum level down to a bacterial species-like level in oligotype profiles of shared bacterial taxa.

## Materials and methods

### Sample collection and DNA extraction

We used fecal samples collected in central Namibia by the cheetah research project (CRP) and the black-backed jackal project (BBJP) of the Leibniz Institute for Zoo and Wildlife Research (IZW) in Berlin, Germany. Amplification of bacterial DNA was possible in 68 samples of clinically healthy free-ranging cheetahs and 50 samples of clinically healthy free-ranging black-backed jackals. Fecal samples from cheetahs were collected from the rectum of immobilized animals during health monitoring, whereas fecal samples from black-backed jackals were collected from the rectum of individuals that were dissected after being shot by local farmers or hunters as problem animals. The CRP and the BBJP hold research permits from the Namibian Ministry of Environment and Tourism (MET) and all work has been carried out in accordance to the relevant regulatory standards. Samples were kept cool in a car freezer for transport to the research station, deep frozen in liquid nitrogen and transported to the IZW, where they were stored at −80°C in deep freezers.

We applied a combined approach of mechanic disruption and enzymatic lysis of bacterial cells. Approximately 200 mg of thawed feces were filled into a 2 ml lysis tube (Precellys SK-38) to which 1.4 ml buffer ASL (QIAamp Mini Stool Kit) was added. A precellys homogenizer was used to homogenize individual samples (2 × 5200 rpm for 20 s with 10 s pause). After the centrifugation of the fecal suspension, 1.2 ml of the supernatant was used to proceed with the isolation as recommended by the QIAamp Mini Stool Kit protocol (Qiagen, Hilden, Germany). This kit contains an Inhibitex tablet that absorbs PCR inhibitors which often cause problems when amplifying DNA from fecal isolates. All handling material (sterile scraper and plate, gloves etc.) was exchanged after each single preparation and the workbench was sterilized before the next extraction.

### 16s rDNA library preparation and sequencing

16S rDNA libraries for cheetahs and black-backed jackals were prepared independently but following the same protocol. We used the approach and the chemistry of Fluidigm (Access Array™ System for Illumina Sequencing Systems, © Fluidigm Corporation) in which PCR and barcoding occur simultaneously. The primers 515F (5′-GTGCCAGCMGCCGCGGTAA-3′) and 806R (5′-GGACTACHVGGGTWTCTAAT-3′) which target a 291 bp-fragment of the hypervariable V4 region of the 16S rRNA gene were used for amplification (Caporaso et al., 2012; Kuczynski et al., 2012). These primers had to be modified according to the Fluidigm protocol and thus were tagged with sequences (CS1 forward tag and CS2 reverse tag) which were complementary to the respective forward or reverse access array barcode primers for Illumina. Final concentrations for the 10 μl target specific 4-primer amplicon tagging reaction were 10 ng/μl DNA, 1X FastStart PCR grade nucleotide mix buffer without MgCl_2_ (Roche), 4.5 mM MgCl_2_ (Roche), 200 μM of each PCR grade nucleotide (Roche), 0.05 U/μl FastStart high fidelity enzyme blend (Roche), 1X access array loading reagent (Fluidigm), 400 nM access array barcode primers for Illumina (Fluidigm), 5% DMSO (Roche), 2.4% PCR certified water and 50 nM target specific primers (TS-515F and TS-806R). In a standard PCR machine we ran the samples as described in the manufacture's protocol (Access Array®, Fluidigm 2012, San Francisco, USA). All individually barcoded samples were subsequently purified using SPRI Based Size Selection (Beckman Coulter Genomics, Brea, CA) with a 1:1 ratio of amplicons to beads and quantified with the Quant-iT™ PicoGreen® kit (Invitrogen/Life Technologies, Green Islands, NY). We then pooled all samples with an equal amount of 15 ng of DNA and diluted the pool down to 8 nM in hybridization buffer. Finally, the libraries were sequenced in two different paired-end runs on Illumina® MiSeq.

### Bioinformatics

We applied the same basic bioinfomatic pipeline to all demultiplexed reads from 68 cheetahs and 50 black-backed jackals. Initially, paired-end reads were merged using FLASH (Magoè and Salzberg, 2011) and primers were cut with the software cutadapt (Martin, 2011). Then, we performed a quality filtering (Q30) and converted fastq-files to fasta-files using the FASTX-Toolkit (FASTX-Toolkit)^1^. Subsequently, all individual fasta-files were merged into a single file and used as a starting point for downstream analyses in the “Quantitative Insights Into Microbial Ecology” (QIIME) software package (Caporaso et al., 2010b). Reads were checked for chimera using the UCHIME algorithm implemented in USEARCH 6.1. Afterwards, reads were pre-clustered at 60% identity against the reference data base using PyNast (Caporaso et al., 2010a). Any reads that failed to hit were discarded. For designation of OTUs, we followed the generally accepted similarity threshold of 97% (Muegge et al., 2011; Caporaso et al., 2012; Bermingham et al., 2013) and applied an open-reference OTU-picking approach using the USEARCH algorithm (Edgar, 2010; Edgar et al., 2011). Thus, besides OTUs which consisted of reads that were clustered against the Greengenes database (version 13.5, http://greengenes.lbl.gov), the remaining reads were clustered into OTUs *de novo* because they did not hit the reference sequence collection. Subsequently, singletons were removed and taxonomy was assigned using the ribosomal database project (RDP) classifier with a minimum confidence to record assignment set at 0.8 (Wang et al., 2007). Finally, reads were cleaned of any non-bacterial ribosomal reads. Alpha diversity for cheetahs and black-backed jackals was calculated on sub-samples of 8000 reads per individual to eliminate the differences in sequencing effort between species and individuals. We calculated (1) the OTU abundance, (2) the Shannon index, which is widely used to calculate diversity based on the number of different data categories and their respective abundance in a data set (Shannon and Weaver, 1949; Spellerberg and Fedor, 2003), and (3) phylogenetic diversity (PD), which is the sum of the branch lengths for all taxa that are part of a given sample (Faith, 1992). We compared alpha diversity measures between cheetahs and black-backed jackals using the Wilcoxon rank sum test. To estimate to which extent the total alpha diversity of an individual was sampled, we plotted the accumulation of Shannon index and PD against sampling effort (number of reads) for each individual (Supplementary Figure 1). Because some bacterial taxa were only present in cheetahs and others only in black-backed jackals we tested whether the proportions of taxa differed significantly between the two species using a Kolmogorov-Smirnov (K-S) test with 1000 bootstraps to calculate the *p*-value [“ks.boot” function in R package “Matching” (Sekhon, 2011)]. The K-S test only compares the similarity in sample diversity but does not account for community composition. Therefore, we calculated beta diversity on a subset (8000 reads) of each cheetah and black-backed jackal microbiome using the unweighted UniFrac metric (Lozupone and Knight, 2005; Lozupone et al., 2011) and applied a PERMANOVA approach (“adonis” in R package “vegan”). We tested the significance of the differences in community composition with a permutation test with 1000 permutations. In addition, we calculated the mean Bray-Curtis distance in cheetahs and black-backed jackals separately (Bray and Curtis, 1957).

We applied oligotyping on all reads which were assigned to a bacterial taxon that was shared between cheetahs and black-backed jackals (*Bacteroides, Blautia, Clostridium, Collinsella, Dorea, Enterococcus*, [*Eubacterium*], *Lactobacillus, Megamonas, Parabacteroides, Peptococcus, Peptostreptococcus, Phascolarctobacterium*, [*Prevotella*], *Ruminococcus*, [*Ruminococcus*], *Slackia, SMB53, Streptococcus, Sutterella*). Because most reads assigned to *Enterobacteriaceae* and *Fusobacteriaceae* were not resolved any better, they were extracted from the family level. To compare oligotype profiles between cheetahs and black-backed jackals, we pooled reads of shared bacterial taxa according to host species for further analyses. Taxa in squared brackets such as [*Ruminococcus*] are recommended groupings by Greengenes database managers based on whole genome phylogeny. However, they are not officially recognized groupings according to Bergey's manual of determinative bacteriology (Bergey et al., 1975) based on physiochemical and morphological traits. Reads were aligned against the Greengenes “core_set_aligned.fasta.imputed” alignment using PyNAST (Caporaso et al., 2010a). We stripped common gaps from each alignment and rarefied both samples (cheetah and black-backed jackal) within each alignment depending on the maximum shared sequence abundance. Subsequently, we conducted the entropy analysis which is based on the Shannon entropy for each base position in the alignment. Oligotyping (version 0.96; http://oligotyping.org) was performed using as many highly variable base positions as necessary to resolve all oligotypes in a bacterial taxon. If a taxon had 200,000–50,000 reads per sample (cheetah and black-backed jackal), an oligotype was considered true if it occurred in more than 1% of all reads (*a* = 1), if the most abundant unique sequence occurred at a minimum of 50 reads (*M* = 50) and if the minimum actual abundance of an oligotype in both samples was more than 500 reads (*A* = 500). For taxa that had less reads per sample (50,000-30,000; 30,000-10,000; 10,000-750) we downsized the *M*-value (25; 10; 5) and the *A*-value (250; 50; 10), respectively. In addition, we applied oligotyping also on the individual level to all bacterial taxa for which oligotype profiles were ≥75% dissimilar in the between-species comparison. We excluded *Lactobacillus, Megamonas*, and *Parabacteroides* from this analysis due to the limited number of host individuals which contributed to this bacterial taxon. Because sequencing depth differed between host species and proportions of bacterial taxa differed naturally between individuals, parameters for oligotyping on the individual level differed from the between-species comparison. In order to treat all samples equally, we only applied the minimum percent abundance parameter (*a* = 5%, *A* = 0, *M* = 0).

We tested whether the number of oligotypes differed significantly between cheetahs and black-backed jackals by comparing the numbers of oligotypes found for each species and for each taxon against each other, again using the ks.boot function (R-package “Matching”; 1000 bootstraps). Furthermore, we measured the dissimilarity in oligotype profiles as the average proportion of reads that differed between cheetahs and black-backed jackals across oligotypes for each bacterial taxon. We then tested whether these average proportions differed between cheetahs and black-backed jackals using a permutation test where we compared observed average proportions to those obtained by randomly assigning each sequence to either one or the other carnivore species with the same probability (0.5). For visualization of heatmap, alpha diversity measures and oligotype barplots we used the packages “phyloseq” (McMurdie and Holmes, 2013) and “ggPlot2” (Wickham, 2009) in R. The principal coordinate analysis (PCoA) plot was produced in QIIME (Caporaso et al., 2010b). All statistical analyses were conducted in R 3.0.2 (R Core Team, 2013). Sequencing data is deposited at the Sequence Read Archive (SRA) under the accession number SRP044660.

## Results

Initially, our next generation sequencing approach of the hypervariable V4 region of the 16S rRNA gene provided 6,117,462 reads for 68 cheetahs and 1,757,276 for 50 black-backed jackals. The number of reads was higher in cheetahs because black-backed jackals were sequenced together with other projects in one Illumina MiSeq run which decreased the number of reads per sample. To account for this bias in number of reads between the two species, all diversity estimates were calculated based on a sub-sampling of 8000 reads per individual. After all initial quality filtering steps were applied to prepare reads for further analyses in QIIME, we proceeded with 5,339,319 reads (87.3%) for cheetahs and 1,344,632 reads (76.5%) for black-backed jackals with an average read length of 252 bp. The open-reference OTU picking resulted in 4033 OTUs which consist of reads that were clustered against the Greengenes database. In addition, 16,271 OTUs were picked *de novo* because associated reads did not hit the reference sequence collection. Rarefaction analyses based on the Shannon index and PD revealed that the sequencing effort was sufficient to describe and compare bacterial communities within and between the two species (Supplementary Figure 1).

**Figure 1:**
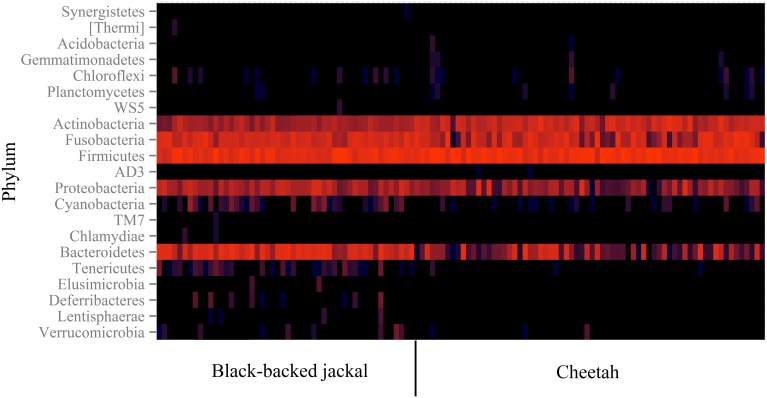
**Heatmap of 16S rRNA gene reads assigned to taxonomy on the bacterial phylum level**. The color encodes the abundance of OTUs (log-scale of base 4) allocated to a specific phylum. Each column represents data of an individual black-backed jackal (left) or cheetah (right) based on a sub-sampling of 8000 reads per individual. The two species are similar in their phylum profiles and share all phyla with proportions above 0.2%.

Some reads could not be assigned to a phylum and thus remained on the kingdom level of bacteria (0.5% reads of cheetahs and 0.3% reads of black-backed jackals). Basically, cheetahs and black-backed jackals had the same most abundant (>0.2%) bacterial phyla (Figure 1). Cyanobacteria and Tenericutes were only represented in the black-backed jackal with proportions ≥0.1% (0.2 and 0.1%, respectively). Differences in proportions of bacterial phyla were pronounced between the species for Actinobacteria (15.5% cheetah vs. 3.8% black-backed jackals), Bacteroidetes (5.8% cheetah vs. 26.1% black-backed jackals) and Firmicutes (56.2% cheetah vs. 40.5% black-backed jackals). Cheetahs and black-backed jackals had these phyla in common with domestic cats, dogs, and other carnivores (Table 1).

**Table 1:**
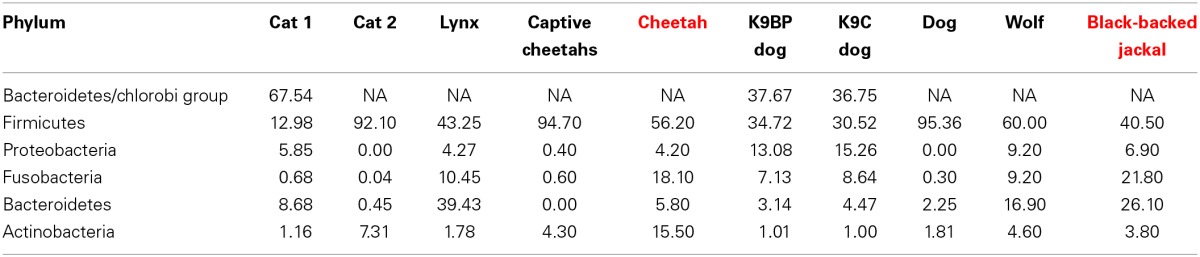
**Proportions (%) of dominant phyla present in domestic cats (cat 1: Tun et al., 2012; cat 2: Handl et al., 2011), free-ranging Iberian lynx (*Lynx pardinus*, Alcaide et al., 2012) and captive cheetahs (Becker et al., 2014), domestic dogs (K9BP dog and K9C dog: Swanson et al., 2010; dog: Handl et al., 2011) and free-ranging wolf (*Canis lupus*, Zhang and Chen, 2010) and profiles for cheetah (red) and black-backed jackal (red) of this study**.

Bacterial reads of both species were assigned to 74 taxa with some being present in either one or both species with a proportion ≥0.1% at the finest resolution (Supplementary Table 1). Out of these, the black-backed jackal was represented in 68 taxa, whereas the cheetah was represented in only 42 taxa. Overall, the two species shared 37 taxonomic assignments (Supplementary Table 1). On the genus level, *Clostridium* (24.5%), *Collinsella* (12.2%), and *Blautia* (8.9%) were the taxa with the highest proportions in cheetahs, whereas in black-backed jackals *Bacteroides* (15.1%), *Clostridium* (9.2%), and *Fusobacterium* (8.4%) had the highest proportions.

**Figure 2:**
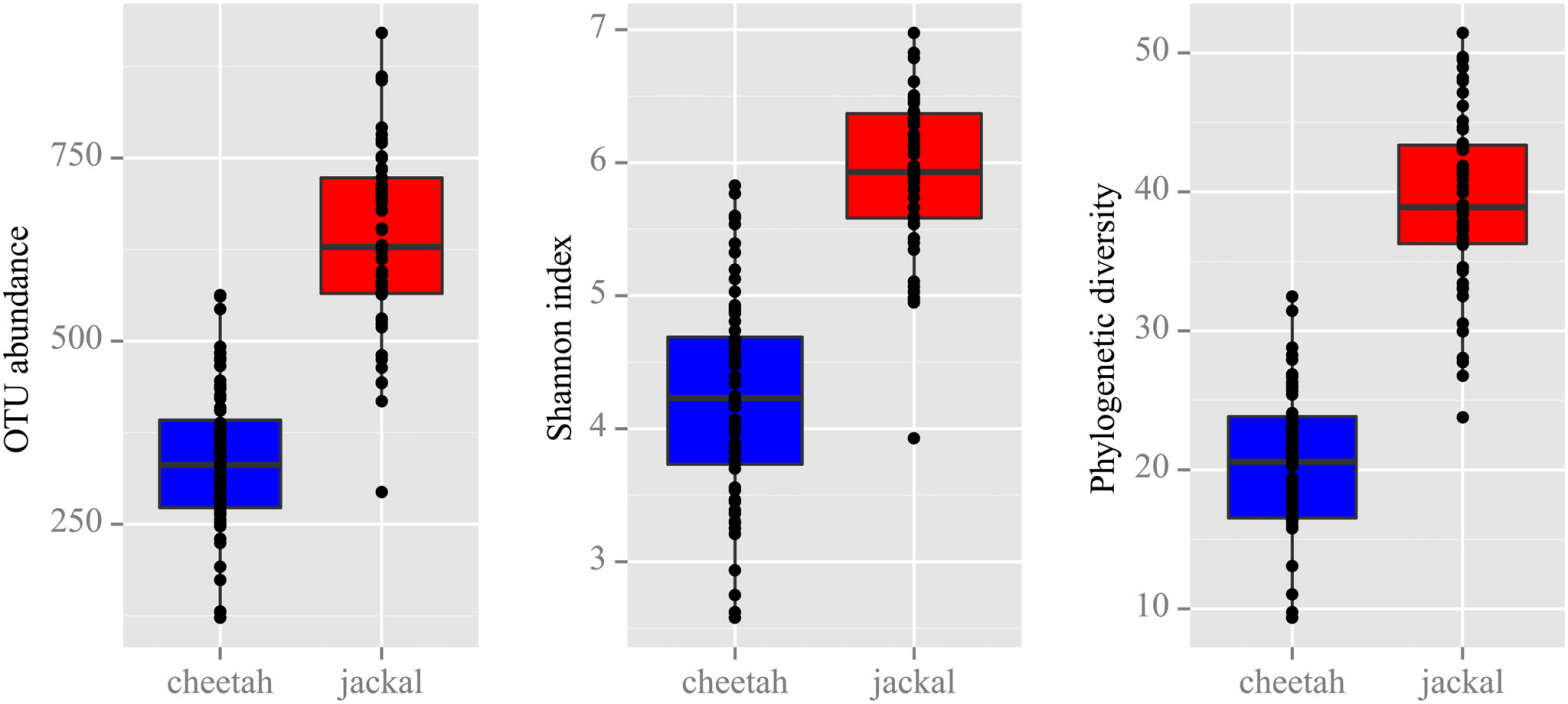
**Alpha diversity measures based on the rarefaction of 8000 reads revealed that black-backed jackals are significantly more diverse than cheetahs for OTU abundance, Shannon index and phylogenetic diversity (Wilcoxon rank sum test: all *p* < 0.001)**.

**Figure 3:**
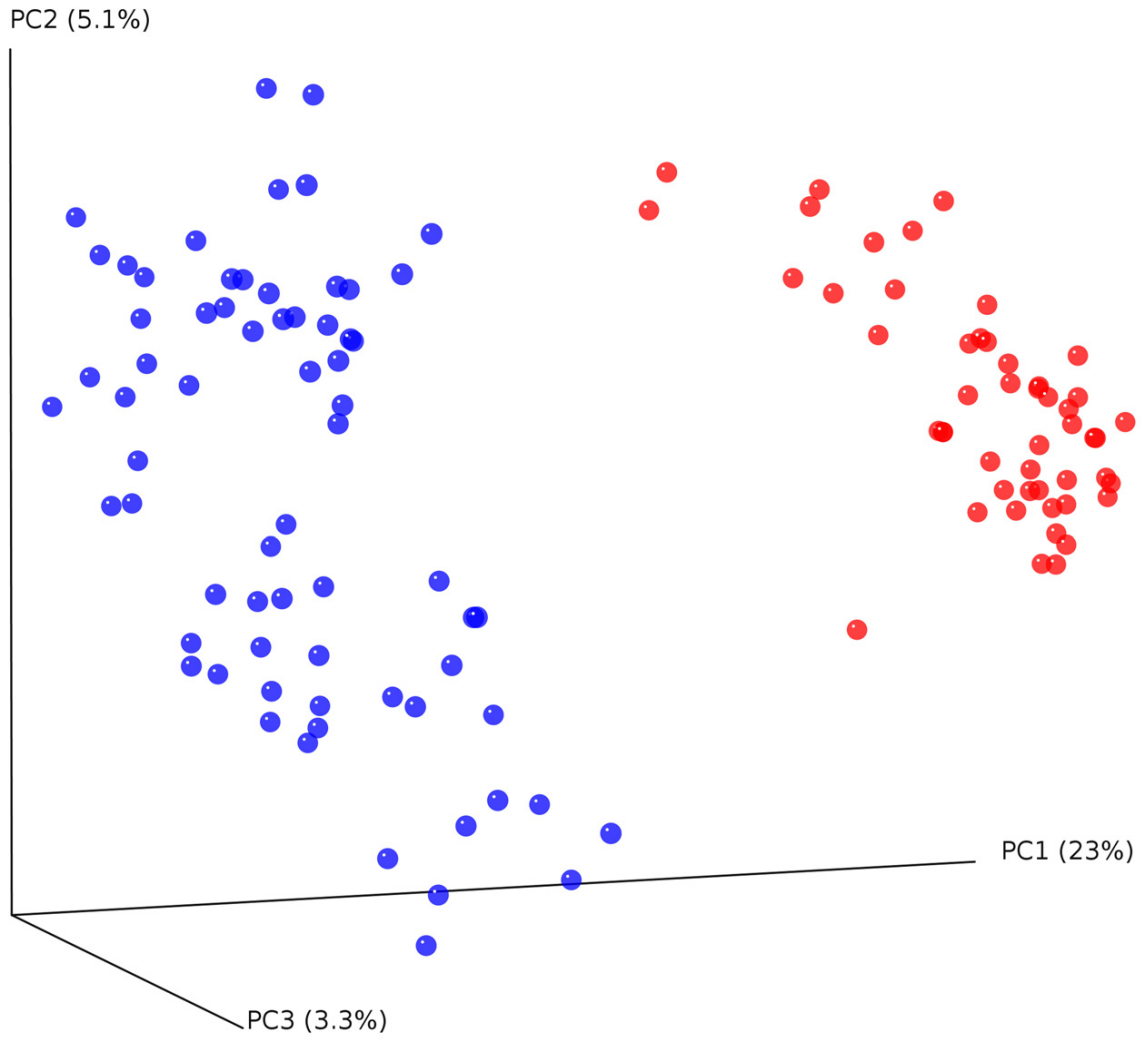
**Principal coordinate analysis (PCoA) plot of cheetahs (blue) and black-backed jackals (red) based on unweighted UniFrac metric**. The two host species differ significantly in their bacterial communities [PERMANOVA: *R*^2^ = 0.74, *F*_(1, 116)_ = 337,02, *p* < 0.001].

To determine whether cheetahs and black-backed jackals can be distinguished from each other based on the diversity of their bacterial communities, we calculated alpha diversity using OTU abundance, Shannon index and PD (Figure 2). OTU abundance was higher in black-backed jackals than in cheetahs (Wilcoxon rank sum test: *W* = 111.5, *p* < 0.001), black-backed jackals had a more diverse bacterial community than cheetahs as revealed by the Shannon index (*W* = 141, *p* < 0.001) and bacterial communities were ecologically more diverse when incorporating information on bacterial phylogeny (*W* = 38, *p* < 0.001). Also, the proportions of bacterial taxa between cheetahs and black-backed jackals were significantly different (ks.boot test (1000 permutations): *D* = 0.35, *p* < 0.01). Beta diversity calculated using the UniFrac metric, which also incorporates bacterial taxonomy, revealed a strong discrimination between the two species [Figure 3; PERMANOVA: *R*^2^ = 0.74, *F*_(1, 116)_ = 337,02, *p* < 0.001]. The three axes of the three-dimensional PCoA plot based on UniFrac distance measures explained more than 30% of the variation in the data set. The Bray-Curtis distance matrix revealed that cheetahs were less similar among each other than black-backed jackals (mean Bray-Curtis similarity indices for cheetahs = 34, for black-backed jackals = 40).

**Figure 4:**
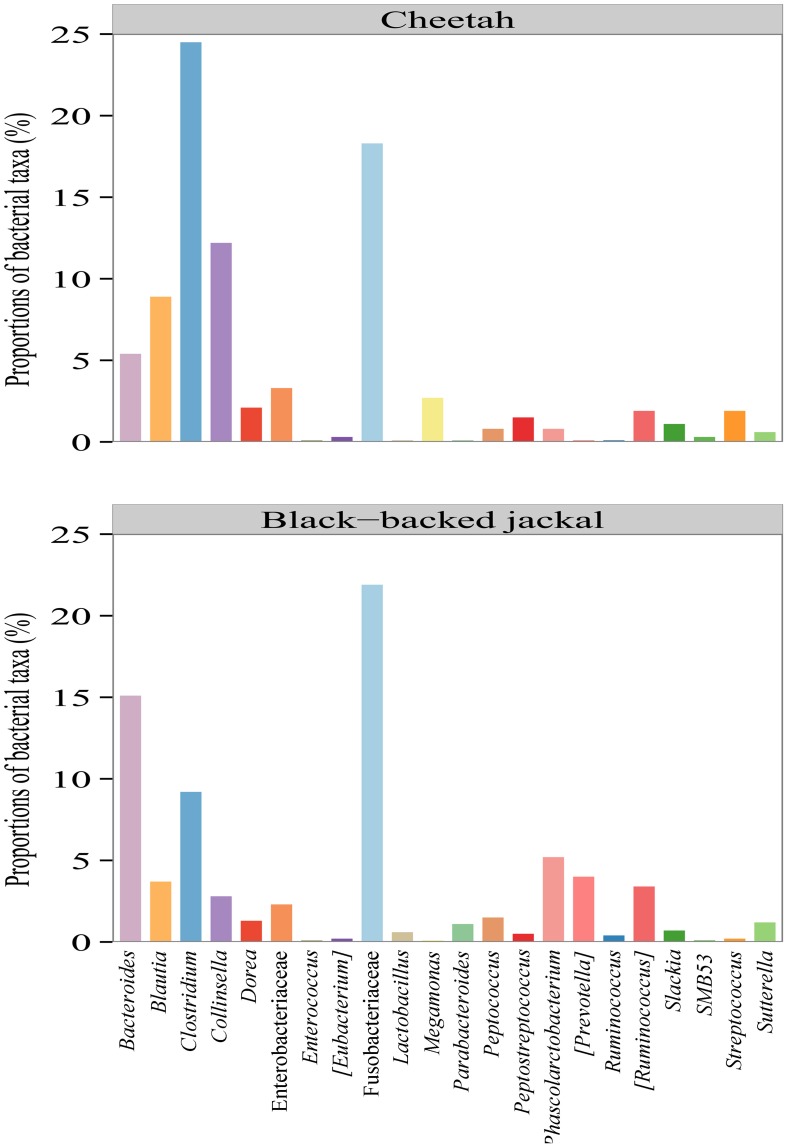
**Proportions of bacterial taxa which were present in both the cheetah and the black-backed jackal with proportions ≥0.1% (pooled on host species level) at the finest taxonomic resolution**. Oligotyping was performed on all 20 bacterial genera and the two bacterial families to resolve the within taxon variation of bacteria between host species on a bacterial species-like level.

**Figure 5:**
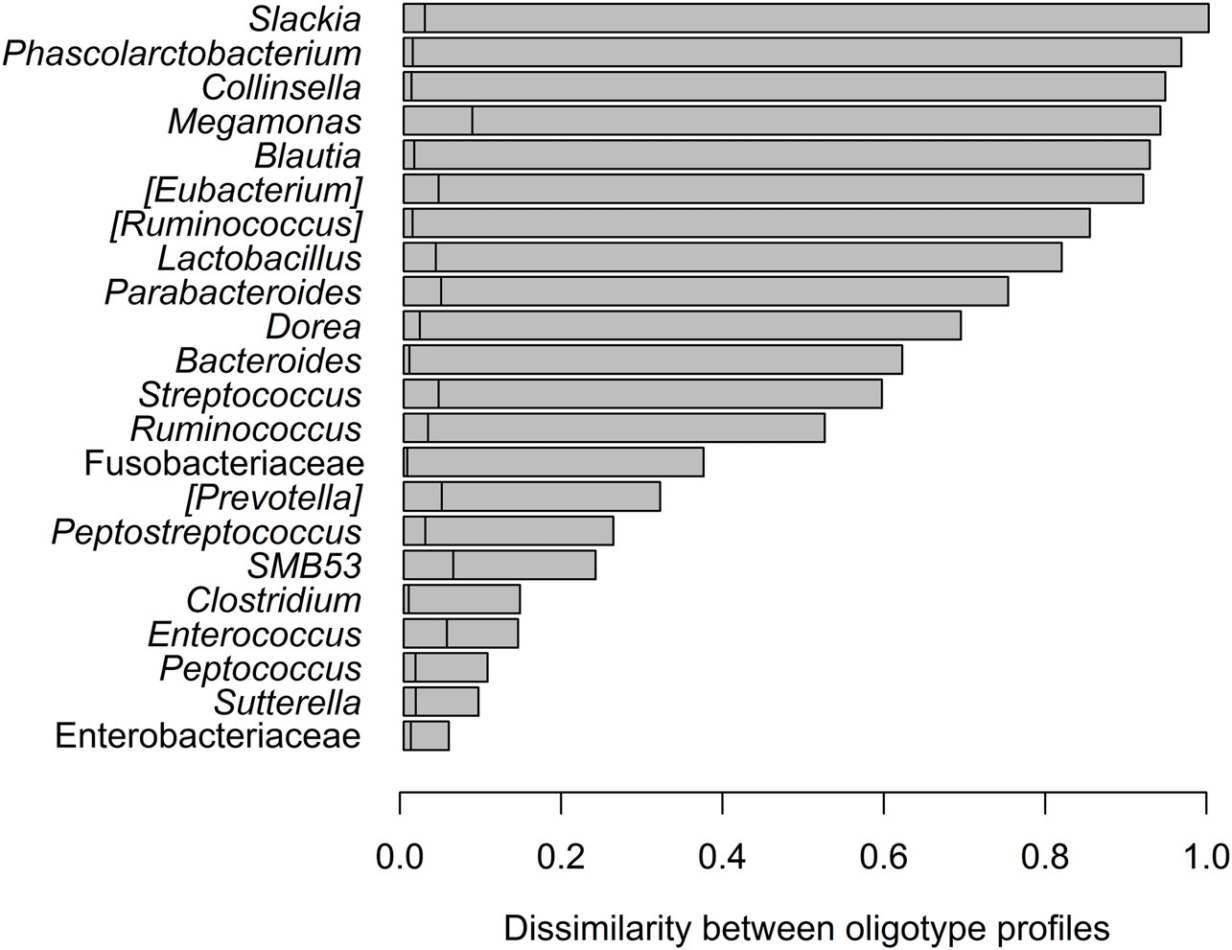
**Dissimilarity in oligotype profiles for each bacteria taxon shared between cheetahs and black-backed jackals**. Observed dissimilarities are ranked from highest (top) to lowest differences (bottom). The vertical lines in each horizontal bar represent the 95% quantile above which differences between the observed and randomized dissimilarities become statistically significant. All observed dissimilarities were higher than randomized ones (*p* < 0.001).

**Figure 6:**
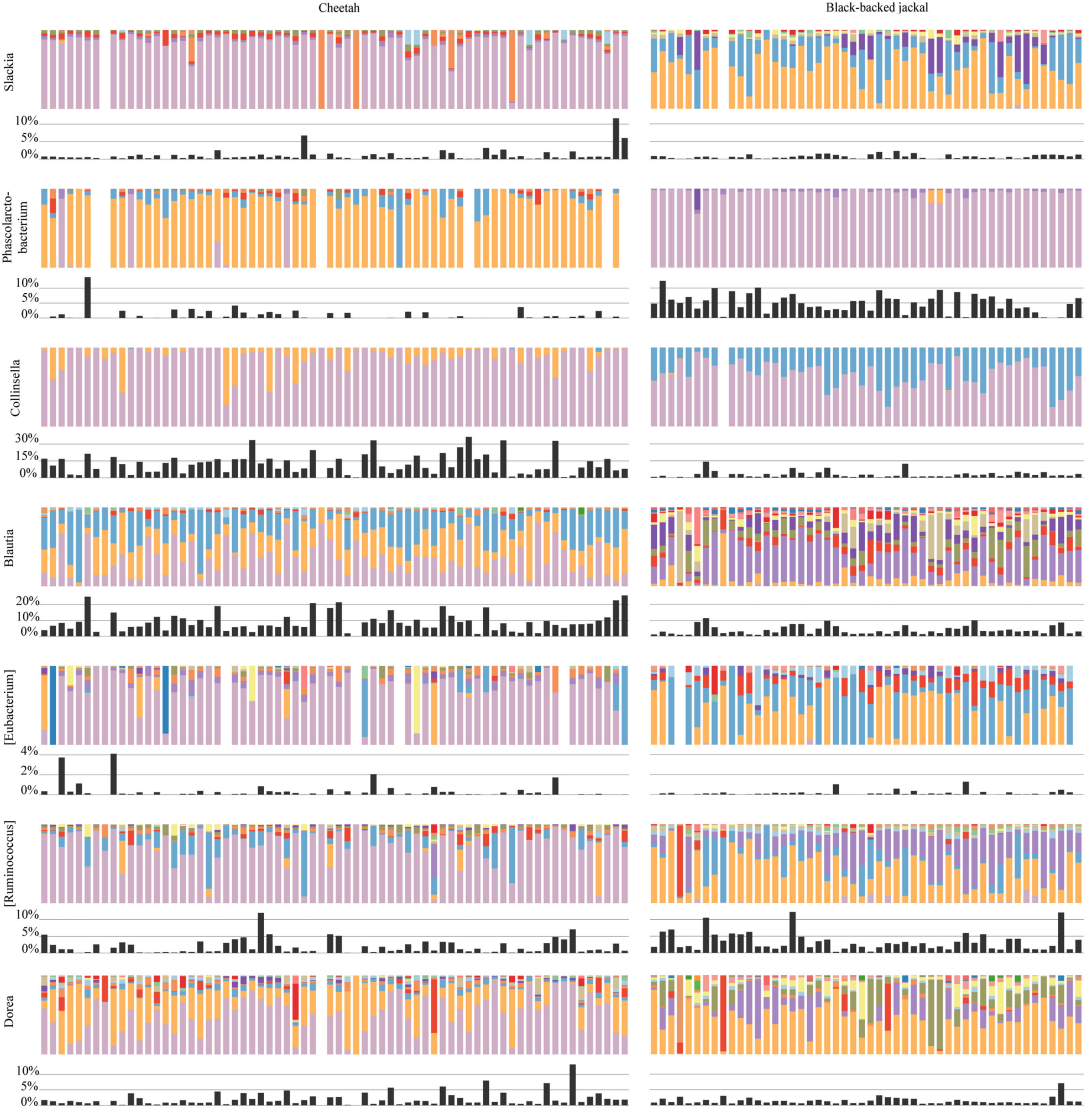
**Oligotype profiles for each cheetah and black-backed jackal individual for shared bacterial taxa which were ≥75% dissimilar in the species level comparison**. *Lactobacillus, Megamonas*, and *Parabacteroides* are excluded from this analysis due to the limited number of host individuals which contributed to this bacterial taxon. Colors of bars within but not between bacterial taxa reflect the same oligotype and the respective proportion present in each individual. Black bars show the proportions of reads that were assigned to each bacterial taxon for every individual.

Oligotyping of bacterial reads extracted from 20 shared genera and two shared families (Figure 4) revealed differences in representative oligotypes and oligotype diversity between cheetahs and black-backed jackals (Table 2, Supplementary Table 2). *Collinsella* and *Lactobacillus* were the genera with the lowest and highest number of oligotypes, respectively, in both species. In general, black-backed jackals had a higher number of oligotypes for 12 out of 22 taxa, particularly in *Blautia* and *Megamonas*. Only in the genera [*Eubacterium*] and *Phascolarctobacterium* cheetahs carried a higher number of oligotypes. In *Clostridium, Enterococcus, Enterobacteriaceae, Fusobacteriaceae, Peptococcus, Peptostreptococcus*, [*Ruminococcus*], and *Sutterella* the number of oligotypes were identical in both species. Within each genus and within the two families of bacteria the proportions of shared oligotypes varied substantially between cheetahs and black-backed jackals, and some oligotypes were exclusively found in one species (Supplementary Figure 2). In 60% of cases the oligotype with the highest proportion was different for both species (Supplementary Figure 2). Overall, the distribution of number of oligotypes per taxon did not differ significantly between cheetahs and black-backed jackals [K-S test (1000 permutations): *D* = 0.18, *p* = 0.69]. The observed differences in proportions of oligotypes between the two species strongly varied between bacterial taxa (Figure 5). The genus *Slackia* exhibited the highest oligotype differences, whereas the family *Enterobacteriaceae* only showed a weak differentiation between the host species. Observed differences differed strongly from a random assignment of oligotypes to cheetah and black-backed jackal within each bacterial taxon (Figure 5; randomization test: *p* < 0.001). When we applied the oligotyping approach on the level of cheetah and black-backed jackal individuals, results were in line with the species-level comparison and individuals exhibited strong differences in oligotype profiles according to species identity (Figure 6). Nevertheless, results from oligotyping on the level of species and individuals are only comparable in a qualitative but not in a quantitative way due to differences in the data sets and parameters which were used for oligotyping.

**Table 2:** **Oligotyping results for the cheetah and the black-backed jackal for shared bacterial genera and families**.

**Taxon**	**Initial reads/after filter**	**Reads after filter cheetah/jackal**	**OT in cheetah**	**OT in jackal**	**Number of shared OT**	**Base positions required to resolve OT**
**GENUS**						
*Bacteroides*	400,000/285,663	154,366/131,297	16	17	16	30
*Blautia*	99,000/81,059	41,554/39,505	6	17	6	21
*Clostridium*	246,200/219,461	109,061/110,400	12	12	12	9
*Collinsella*	75,800/73,271	36,696/36,575	5	6	5	5
*Dorea*	35,000/29,436	14,862/14,574	10	12	7	15
*Enterococcus*	3100/2606	1295/1311	10	10	10	16
*[Eubacterium]*	6000/5418	2755/2663	11	9	7	13
*Lactobacillus*	12,000/9719	5151/4568	17	19	12	20
*Megamonas*	1500/1231	633/598	4	10	4	14
*Parabacteroides*	8600/6539	3363/3176	13	16	12	35
*Peptococcus*	39,000/36,792	18,237/18,555	9	9	9	5
*Peptostreptococcus*	13,200/12,508	6233/6275	10	10	10	8
*Phascolarctobacterium*	74,000/64,533	31,905/32,628	10	8	8	17
*[Prevotella]*	6300/5157	2682/2475	14	15	14	15
*Ruminococcus*	10,700/9255	4700/4485	6	9	6	25
*[Ruminococcus]*	89,400/78,556	40,974/37,582	10	10	10	10
*SMB53*	3300/2756	1385/1371	11	12	11	14
*Slackia*	18,000/16,058	8458/7627	9	10	4	15
*Streptococcus*	5400/5168	2605/2563	7	11	6	7
*Sutterella*	31,800/29,494	14,810/14,684	8	8	8	9
**FAMILY**						
Enterobacteriaceae	31,859/31,642	24,430/24,264	7	7	7	4
Fusobacteriaceae	581,800/497,644	263,944/233,700	15	15	15	16

*Reads were extracted from the respective bacterial taxon to which they were assigned to and sub-sampled according to the maximum number of shared reads. After filtering, the remaining reads were used for oligotyping with the minimum number of base positions required to resolve all oligotypes (OT) in both samples*.

## Discussion

Gut-associated microbial communities of two free-ranging Namibian carnivore species vary increasingly from the bacterial phylum level down to the bacterial species-like level of oligotypes. In general, most bacterial phyla to which reads were assigned were shared among both species, whereas the phyla Cyanobacteria and Tenericutes were only present in black-backed jackals with proportions ≥0.1%. The phylum Tenericutes, especially the genus *Mollicutes*, comprises many parasitic bacteria (e.g., *Mycoplasma canis*) which can cause urogenital tract diseases (Chalker, 2005; Waltzek et al., 2012). Cyanobacteria are present in many terrestrial habitats but they are also very abundant in aquatic habitats (Stanier and Bazine, 1977). Thus, black-backed jackals might carry a higher proportion of Cyanobacteria because they also feed on amphibians (Walton and Joly, 2003). In addition, the proportions of shared bacterial genera were different between the two species. Black-backed jackals had higher proportions of *Bacteroides* and [*Prevotella*] than cheetahs. These two genera are known to be influenced by the diet (David et al., 2013). *Bacteroides* is associated with animal protein, several amino acids and saturated fats, whereas *Prevotella* is associated with hemicelulose and simple carbohydrates (Wu et al., 2011). Thus, the omnivorous diet of black-backed jackals requires a higher proportion of *Prevotella* to digest also plant material. In contrast, cheetahs would be expected to harbor a higher proportion of *Bacteroides* because of their strict carnivorous diet, yet this was not the case in our study. Furthermore, the proportions of the genera *Blautia, Clostridium, Megamonas*, and *Peptostreptococcus* increased when hosts fed on a diet with high fat contents compared to a diet with low fat contents (Bermingham et al., 2013). This relationship was reversed for *Lactobacillus* spp. which are usually seen as a beneficial group of microbes supporting nutrient acquisition in herbivores (Famularo et al., 2005). In the present study, *Lactobacillus* had a higher proportion in the omnivorous black-backed jackal, whereas the other mentioned genera had higher proportions in the cheetah. Thus, our findings suggest that a strictly carnivorous diet leads to a higher fat intake than an omnivorous diet.

Microbiomes of cheetahs and black-backed jackals share some characteristics with microbiomes of domestic cats and dogs (Swanson et al., 2010; Handl et al., 2011; Tun et al., 2012) and other mammals (Ley et al., 2008; Zhang and Chen, 2010; Alcaide et al., 2012). The microbiomes differ mainly in proportions of phyla rather than differences in diversity (Table 1). In one of the first studies on gut-microbial communities using 454 next-generation sequencing in domestic cats and dogs, the phylum with the highest proportions was Firmicutes followed by Actinobacteria in cats and Bacteroidetes in dogs (Handl et al., 2011). Black-backed jackals harbor the same taxa in high proportions as dogs, whereas for cheetahs the second most abundant phylum was Fusobacteria followed closely by Actinobacteria. When looking at a higher taxonomic resolution, cheetahs and black-backed jackals shared the same genera (*Slackia* and *Collinsella*) within the phylum Actinobacteria which was also true for domestic dogs but not for domestic cats that carried *Eggerthella* and *Olsenella* (Handl et al., 2011). To our knowledge, only two studies focused on the microbiomes of free-ranging wildlife species belonging to the feline (Alcaide et al., 2012) and canine family (Zhang and Chen, 2010). These studies investigated the gut-bacterial communities of free-ranging Iberian lynx (*Lynx pardinus*) and free-ranging wolf (*Canis lupus*), respectively. Cheetahs and Iberian lynx both have high proportions of the phylum Firmicutes (56.20 vs. 43.25%) and similar proportions for the phylum Proteobacteria (4.20 vs. 4.27%). However, differences were quite pronounced for Fusobacteria (18.10 vs. 10.45%), *Bacteroides* (5.80 vs. 39.43%) and Actinobacteria (15.50 vs. 1.78%). Gut-bacterial communities of two captive cheetahs analyzed with 16S rRNA gene clone libraries (Becker et al., 2014) were very different from bacterial communities of free-ranging cheetahs in our study. Captive cheetahs had high proportions of Firmicutes (94.7 vs. 56.2% in free-ranging cheetahs) and low proportions of, e.g., Fusobacteria (0.6 vs. 18.1%). In contrast, black-backed jackals and free-ranging wolves exhibited similar ranks for bacterial phyla. Nevertheless, proportions also differed between Firmicutes (40.50 vs. 60.00%), Fusobacteria (21.80 vs. 9.20%) and *Bacteroides* (26.10 vs. 16.90%). Although these findings might be biased to some extent by the varying extraction and sequencing methods and differences in samples sizes, they reveal large variation in bacterial proportions already at the phylum level.

Comparisons between cheetahs and black-backed jackals using the alpha diversity measures OTU abundance, Shannon index and PD revealed that black-backed jackals had higher alpha diversities for all measures. Also, beta diversity measures based on UniFrac and Bray-Curtis distance clearly discriminated bacterial diversity according to host species. The microbial community in the black-backed jackal needs to achieve digestion of prey items from various sources ranging from meat to plant material, whereas bacteria in cheetahs are confronted with a much more restricted diet. In addition, the social system of black-backed jackals and their foraging behavior favors the exchange of bacteria via contact with conspecifics and intake of bacteria from carcasses (Walton and Joly, 2003; VanderWaal et al., 2013). The fact that cheetahs use a larger territory promoting microbial intake from a wide range of habitat types seems to be of minor impact compared to the factors that drive bacterial diversification in black-backed jackals. Although evidence exists that individuals exhibit different microbiomes in geographically distant habitats, differences have only been demonstrated for within-species comparisons (Fallani et al., 2010; Linnenbrink et al., 2013). When looking at between-species differences, environmental factors are difficult to distinguish from other factors such as host phylogeny, behavior or diet (Ley et al., 2008; Phillips et al., 2012).

Assignment of taxonomy to bacterial OTUs is a common approach to investigate bacterial taxa present in samples of interest. Nevertheless, due to the restrictions in resolution and richness of bacterial databases, assignments are rarely better than genus level. To resolve bacteria within the same genus between host species requires a higher taxonomic resolution. We have achieved this by oligotyping reads extracted from shared bacterial taxa in cheetahs and black-backed jackals. Thereby, we revealed a strong association and differentiation of oligotypes according to host species which was not explained by the OTU-clustering approach. Some genera exhibited strong differences in oligotype profiles, whereas others were similar with changes only in proportions of oligotypes. These differences in oligotypes might be due to a co-evolutionary fine-tuning of some genera according to the digestive requirements of the host (Ley et al., 2008). Alternatively, they may reflect the bacterial “speciation” in an enclosed host system in which almost no genetic exchange exists with the respective bacteria in another host species. Although oligotypes are different between the two carnivores, a functional redundancy might enable them to possess similar microbial genes and metabolic pathways (Suchodolski et al., 2009; Muegge et al., 2011). In most taxa one oligotype accounted for the majority of bacterial reads, which demonstrates that the diversity of genera might be more important for the digestive requirements of a host than the bacterial diversity within a genus.

## Conclusion

Oligotyping in our study revealed gut microbiome differences between the cheetah and the black-backed jackal at a high taxonomic resolution. As a technique, oligotyping encompasses the limitation of the OTU approach by decomposing bacterial OTUs or taxa by minimizing the entropy within a group of reads down to a single base. Thus, resolved oligotypes act as proxies for bacterial species and thereby increase the amount of in-depth information that can be extracted from short sequencing reads of the 16S rRNA gene. By applying this new approach in conjunction with the OTU clustering approach we described similarities and differences between these two carnivore species from the bacterial phylum down to the species-like level of oligotypes. Cheetahs exhibited a lower bacterial diversity than black-backed jackals indicating that the size of home ranges is less important in shaping the microbiome of a host than the respective diet, foraging behavior and social system.

## Author contributions

Conceived and designed the experiments: Sebastian Menke, Simone Sommer. Performed the experiments: Sebastian Menke, Matthias Meier, Wasimuddin. Field logistic and sample collection: Sebastian Menke, Matthias Meier, Jörg Melzheimer, Sonja Heinrich, Susanne Thalwitzer, Bettina Wachter, John K. E. Mfune. Analyzed the genomic data: Sebastian Menke. Statistical analysis: Sebastian Menke. Wrote the paper: Sebastian Menke drafted the manuscript, Simone Sommer, Bettina Wachter, Wasimuddin critically reviewed the manuscript. All authors read and approved the final manuscript.

### Conflict of interest statement

The authors declare that the research was conducted in the absence of any commercial or financial relationships that could be construed as a potential conflict of interest.

## Footnotes

^1^Available online at: http://hannonlab.cshl.edu/fastx_toolkit/index.html by Hannon Lab.
