# Supplementary material for: Oligotyping reveals differences between gut microbiomes of free-ranging sympatric Namibian carnivores (Acinonyx jubatus, Canis mesomelas) on a bacterial species-like level
Source: Front Microbiol. 2014 Oct 14;5:526. doi: 10.3389/fmicb.2014.00526 (PMC4196554; doi:10.3389/fmicb.2014.00526)
Supplement: Supplementary file 1 [file DataSheet1.ZIP › 104677_Sommer_Supplementary_Figure_1.PDF]

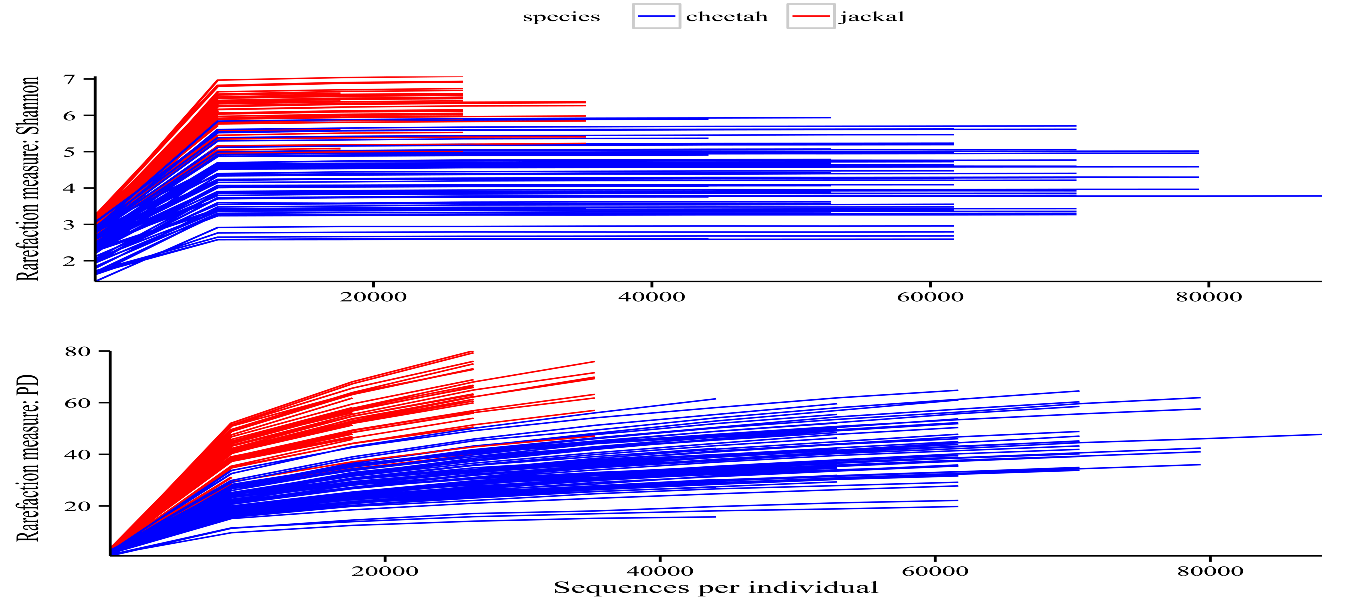

Supplementary Figure 1: Alpha-diversity rarefaction plots for cheetahs (blue) and black-backed jackals (red) starting from 10 up to 88110 sequences with ten steps between minimum and maximum rarefaction depth. Alpha-diversity measures of Shannon index (Shannon) and phylogenetic diversity (PD) were calculated on each rarefied dataset and plotted against the number of rarefied sequences. Rarefaction depths between cheetahs and black-backed jackals differ because respective samples were sequenced in two runs resulting in different numbers of reads.
