# Supplementary material for: Oligotyping reveals differences between gut microbiomes of free-ranging sympatric Namibian carnivores (Acinonyx jubatus, Canis mesomelas) on a bacterial species-like level
Source: Front Microbiol. 2014 Oct 14;5:526. doi: 10.3389/fmicb.2014.00526 (PMC4196554; doi:10.3389/fmicb.2014.00526)
Supplement: Supplementary file 1 [file DataSheet1.ZIP › 104677_Sommer_Supplementary_Figure_2.PDF]

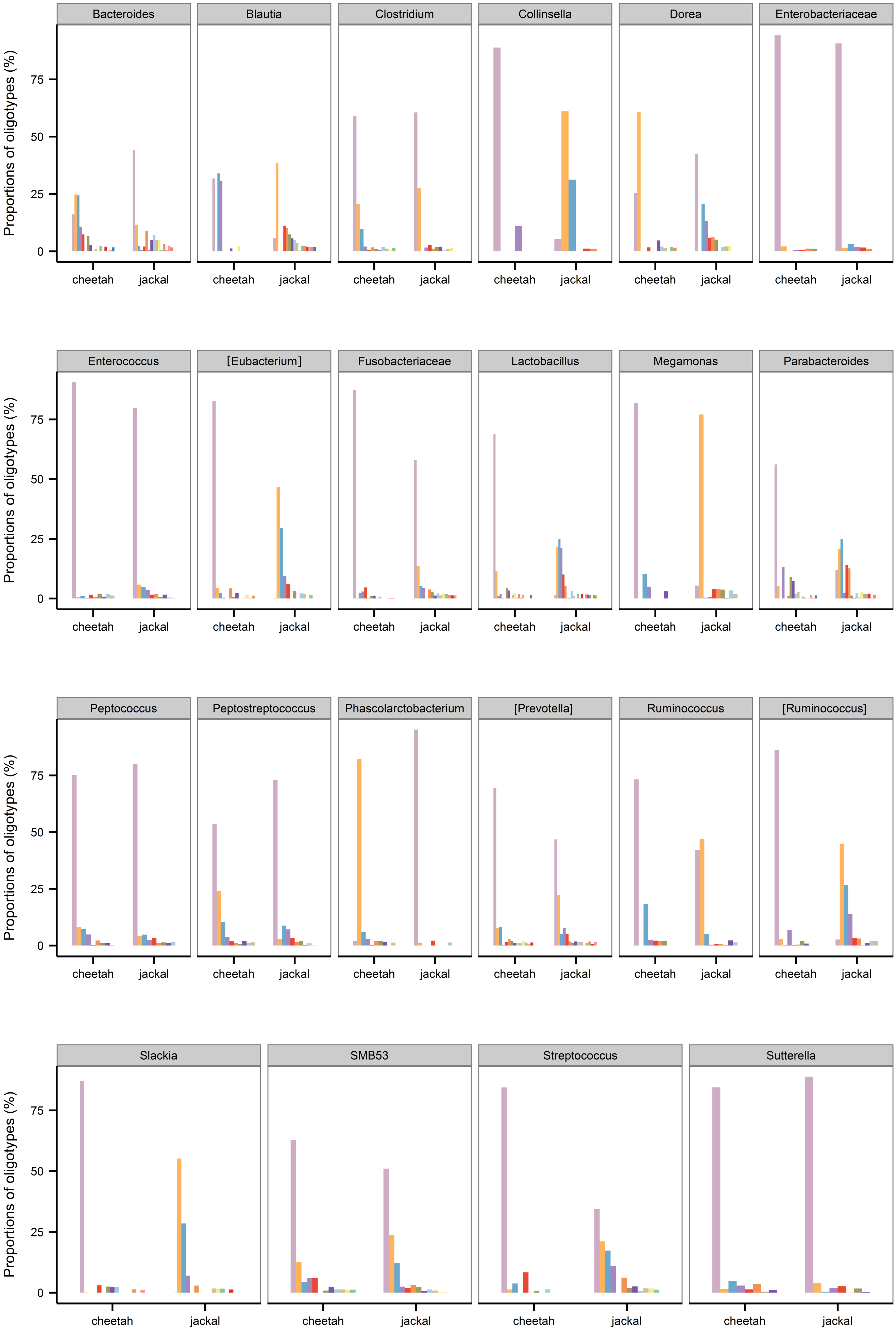

Bacteroides
Blautia
Clostridium
Collinsella
Dorea
Enterobacteriaceae
Enterococcus
[Eubacterium]
Fusobacteriaceae
Lactobacillus
Megamonas
Parabacteroides
Peptococcus
Peptostreptococcus
Phascolarctobacterium
[Prevotella]
Ruminococcus
[Ruminococcus]
Slackia
SMB53
Streptococcus
Sutterella
Proportions of oligotypes (%)
0
25
50
75
cheetah
jackal

Supplementary Figure 2: Oligotype profiles based on proportions of each oligotype within each shared bacterial taxon for cheetahs and black backed jackals. Colors of bars within but not between bacterial taxa reflect the same oligotype.
