# Supplementary material for: Oligotyping reveals differences between gut microbiomes of free-ranging sympatric Namibian carnivores (Acinonyx jubatus, Canis mesomelas) on a bacterial species-like level
Source: Front Microbiol. 2014 Oct 14;5:526. doi: 10.3389/fmicb.2014.00526 (PMC4196554; doi:10.3389/fmicb.2014.00526)
Supplement: Supplementary file 1 [file DataSheet1.ZIP › 104677_Sommer_Supplementary_Table_1.PDF]

Supplementary Table 1: List of bacterial taxa to which OTUs of cheetahs and black-backed jackals were assigned with proportions ≥ 0.1 % at the finest resolution. All taxa that were used for oligotyping are highlighted. “Other” assignments are due to ambiguity when the ribosomal database project (RDP) classifier was not able to decide between distinct taxa, “g_” means that an OTU did match the Greengenes database but that the reference sequence was poorly defined, taxa in brackets are groupings that are suggested by the Greengenes data manager based on whole genome phylogeny but which are not officially recognized according Bergey’s manual of determinative bacteriology (Bergey et al., 1975) based on physiochemical and morphological traits. A “/” means that the proportion of a taxon was below < 0.1 % or not present.

**Taxonomy**	**Cheetah**	**Black-backed-jackal**
k_Bacteria	0.3 %	0.5 %
**Phylum Actinobacteria**		
o_Actinomycetales;Other;Other	0.1 %	/
o_Actinomycetales;f_Micrococcaceae;g_*Micrococcus*	0.1 %	/
o_Coriobacteriales;f_Coriobacteriaceae;Other	1.7 %	/
o_Coriobacteriales;f_Coriobacteriaceae;g_	0.1 %	0.1 %
o_Coriobacteriales;f_Coriobacteriaceae;g_*Collinsella*	12.2 %	2.8 %
o_Coriobacteriales;f_Coriobacteriaceae;g_*Slackia*	1.1 %	0.7 %
**Phylum Bacteroidetes**		
o_Bacteroidales;Other;Other	/	0.5 %
o_Bacteroidales;f_;g_	/	1.0 %
o_Bacteroidales;f_Bacteroidaceae;g_*Bacteroides*	5.4 %	15.1 %
o_Bacteroidales;f_Porphyromonadaceae;g_*Parabacteroides*	0.1 %	1.10%
o_Bacteroidales;f_Prevotellaceae;g_*Prevotella*	/	0.20%
o_Bacteroidales;f_S24-7;g_	0.1 %	0.9 %
o_Bacteroidales;f_[Odoribacteraceae];g_*Odoribacter*	/	0.4 %
o_Bacteroidales;f_[Paraprevotellaceae];Other	/	0.5 %
o_Bacteroidales;f_[Paraprevotellaceae];g_	/	0.6 %
o_Bacteroidales;f_[Paraprevotellaceae];g_*CF231*	/	1.5 %
o_Bacteroidales;f_[Paraprevotellaceae];g_[*Prevotella*]	0.1 %	4.0 %
**Phylum Cyanobacteria**		
o_YS2;f_;g_	/	0.1 %
o_Streptophyta;f_;g_	/	0.1 %
**Phylum Firmicutes**		
p_Firmicutes;Other;Other;Other	/	0.1 %
o_Lactobacillales;Other;Other	/	0.1 %
o_Lactobacillales;f_Enterococcaceae;g_*Enterococcus*	0.1 %	0.1 %

o_Lactobacillales;f_Enterococcaceae;g_*Vagococcus*	/	0.2 %
o_Lactobacillales;f_Lactobacillaceae;Other	/	0.1 %
o_Lactobacillales;f_Lactobacillaceae;g_*Lactobacillus*	0.1 %	0.6 %
o_Lactobacillales;f_Streptococcaceae;g_*Lactococcus*	/	0.1 %
o_Lactobacillales;f_Streptococcaceae;g_*Streptococcus*	1.9 %	0.2 %
o_Clostridiales;Other;Other	0.7 %	0.9 %
o_Clostridiales;f_;g_	/	0.3 %
o_Clostridiales;f_Clostridiaceae;Other	2.8 %	1.2 %
o_Clostridiales;f_Clostridiaceae;g_	0.1 %	0.1 %
o_Clostridiales;f_Clostridiaceae;g_*Clostridium*	24.5 %	9.2 %
o_Clostridiales;f_Clostridiaceae;g_*SMB53*	0.3 %	0.1 %
o_Clostridiales;f_Eubacteriaceae;g_*Pseudoramibacter*	/	0.1 %
o_Clostridiales;f_Lachnospiraceae;Other	2.2 %	2.7 %
o_Clostridiales;f_Lachnospiraceae;g_	/	0.5 %
o_Clostridiales;f_Lachnospiraceae;g_*Blautia*	8.9 %	3.7 %
o_Clostridiales;f_Lachnospiraceae;g_*Coprococcus*	/	0.1 %
o_Clostridiales;f_Lachnospiraceae;g_*Dorea*	2.1 %	1.3 %
o_Clostridiales;f_Lachnospiraceae;g_*Roseburia*	0.1 %	/
o_Clostridiales;f_Lachnospiraceae;g_[*Ruminococcus*]	1.9 %	3.4 %
o_Clostridiales;f_Peptococcaceae;g_*Peptococcus*	0.8 %	1.5 %
o_Clostridiales;f_Peptostreptococcaceae;g_	1.5 %	0.5 %
o_Clostridiales;f_Peptostreptococcaceae;g_*Peptostreptococcus*	1.5 %	0.5 %
o_Clostridiales;f_Ruminococcaceae;Other	0.1 %	1.2 %
o_Clostridiales;f_Ruminococcaceae;g_	/	1.0 %
o_Clostridiales;f_Ruminococcaceae;g_*Faecalibacterium*	/	0.9 %
o_Clostridiales;f_Ruminococcaceae;g_*Oscillospira*	/	0.5 %
o_Clostridiales;f_Ruminococcaceae;g_*Ruminococcus*	0.1 %	0.4 %
o_Clostridiales;f_Veillonellaceae;Other	0.1 %	0.2 %
o_Clostridiales;f_Veillonellaceae;g_*Megamonas*	2.7 %	0.1 %
o_Clostridiales;f_Veillonellaceae;g_*Phascolarctobacterium*	0.8 %	5.2 %
o_Clostridiales;f_[Mogibacteriaceae];g_	0.1 %	2.1 %
o_Clostridiales;f_[Tissierellaceae];g_*Peptoniphilus*	1.9 %	/
o_Erysipelotrichales;f_Erysipelotrichaceae;Other	/	0.1 %
o_Erysipelotrichales;f_Erysipelotrichaceae;g_	/	0.1 %
o_Erysipelotrichales;f_Erysipelotrichaceae;g_*Allobaculum*	/	0.2 %
o_Erysipelotrichales;f_Erysipelotrichaceae;g_[*Eubacterium*]	0.3 %	0.2 %
o_Erysipelotrichales;f_Erysipelotrichaceae;g_*p-75-a5*	/	0.2 %
**Phylum Fusobacteria**		

o_Fusobacteriales;f_Fusobacteriaceae;Other	15.5 %	12.5 %
o_Fusobacteriales;f_Fusobacteriaceae;g_	0.2 %	0.3 %
o_Fusobacteriales;f_Fusobacteriaceae;g_*Cetobacterium*	0.2 %	0.7 %
o_Fusobacteriales;f_Fusobacteriaceae;g_*Fusobacterium*	2.3 %	8.4 %
**Phylum Proteobacteria**		
o_Burkholderiales;Other;Other	/	0.4 %
o_Burkholderiales;f_Alcaligenaceae;g_*Sutterella*	0.6 %	1.2 %
o_Desulfovibrionales;f_Desulfovibrionaceae;g_*Desulfovibrio*	/	0.5 %
o_Campylobacterales;f_Helicobacteraceae;Other	/	0.1 %
o_Campylobacterales;f_Helicobacteraceae;g_*Helicobacter*	/	0.1 %
o_Aeromonadales;f_Succinivibrionaceae;Other	/	0.1 %
o_Aeromonadales;f_Succinivibrionaceae;g_*Anaerobiospirillum*	/	1.9 %
o_Enterobacteriales;f_Enterobacteriaceae;Other	0.4 %	0.3 %
o_Enterobacteriales;f_Enterobacteriaceae;g_	2.9 %	2.1 %
**Phylum Tenericutes**		
k__Bacteria;p_*Tenericutes*	0.0%	0.1%
